# The Details of the Operation of Circumcision

**Published:** 1893-11

**Authors:** J. McF. Gaston

**Affiliations:** Atlanta, Ga.


### THE DETAILS OF THE OPERATION OF CIRCUMCISION.

BY J. McF. GASTON, JR., A. M., M. D., ATLANTA, GA.

The Southern Medical Record of October contains a very interesting report of the method pursued by N. Davies-Colley for the last fifteen years (Guy's Hos, Rep., vol. xlix, 1892). His points are well taken, and, if followed, his method will secure good results and union by first intention all around.

I was especially struck with his idea of leaving a triangular piece of skin to cover the portion of penis below the frænum. It is very annoying to have to wait so long for granulation to fill this space that is usually left by a circular flap, so to speak. I have found also that the new skin becomes hard and unyielding if this point is left unprotected. I think, however that an easier mode of securing this triangular piece can be used than Davies-Colley describes.

I have recently operated as he describes, with this exception, for a very much contracted mucous membrane in a young man, aged 19. Having first been careful to have an assistant hold the mucous membrane forward by means of artery forceps clamped upon the dorsal part of the foreskin where it joins the mucous membrane, the dressing forceps was secured downwards and forwards by an assistant, and the foreskin in front of the dressing forceps, cut with a pair of scissors *curved* downward; and then directly curving the scissors forward, I succeeded in obtaining the triangular piece to fit under the frænum. Then I slit up the mucous membrane to the sulcus back of the corona, and with a pair of straight scissors, cut the mucous membrane, leaving only a frill an eighth of an inch in width. When the stitches were introduced care was taken to secure the triangular piece first, with a silk stitch on each side, and to secure the two pieces of the mucous membrane at the dorsum with silk. The rest of the sutures were catgut, quite a number being placed to connect the triangular piece with the mucous membrane around the frænum. In four days the catgut had been absorbed, but the silk still held, and the union by first intention at the frænum had been secured. In ten days all the stitches had been removed. No discomfort was experienced from an unusual quantity of cocaine which was injected at the time of the operation. A discharge from the penis, that had existed for a year, ceased.

---

MENSTRUAL DERANGEMENT.—Few women are free from those tormenting headaches, backaches, ovarian neuralgias, gastric and vesical irritations during the Menstrual Molimen, and which are so distressing as to drive a much larger number to the use of opium than is generally suspected. Dioviburnia contains no opium, but is equally efficient in giving relief, and at the same time cures by its tonic and alterative effect.

