# Erratum

**Published:** 1893-11

**Authors:** 


ERRATUM—In this issue of the RECORD, page 582, line 10, read *curved* scissors, for scissors *curved*. This is necessary for the understanding of Dr. Gaston Jr.'s method of Circumcision.

